# Estimating the Consequences of Fire Exclusion for Food Crop Production, Soil Fertility, and Fallow Recovery in Shifting Cultivation Landscapes in the Humid Tropics

**DOI:** 10.1007/s00267-014-0431-7

**Published:** 2014-12-24

**Authors:** Lindsey Norgrove, Stefan Hauser

**Affiliations:** 1 Department of Environmental Sciences (Biogeography), University of Basel, St. Johanns Vorstadt 10, 4056 Basel, Switzerland; 2 International Institute of Tropical Agriculture, Oyo Road, PMB 5320, Ibadan, Oyo State Nigeria

**Keywords:** Carbon, Congo Basin, Fire, Land sparing versus land sharing, Maize, Plantain, Soil fertility, Swidden

## Abstract

In the Congo Basin, smallholder farmers practice slash-and-burn shifting cultivation. Yet, deliberate burning might no longer be sustainable under reduced fallow scenarios. We synthesized data from the Forest Margins Benchmark Area (FMBA), comprising 1.54 million hectares (ha), in southern Cameroon and assessed the impact of fire exclusion on yield, labor inputs, soil fertility, ecosystem carbon stocks, and fallow recovery indicators in two common field types (plantain and maize) under both current and reduced fallow scenarios. While we could not distinguish between impacts of standard farmer burning practice and fire exclusion treatments for the current fallow scenario, we concluded that fire exclusion would lead to higher yields, higher ecosystem carbon stocks as well as potentially faster fallow recovery under the reduced fallow scenario. While its implementation would increase labor requirements, we estimated increased revenues of 421 and 388 US$ ha^−1^ for plantain and maize, respectively. Applied to the FMBA, and assuming a 6-year reduced fallow scenario, fire exclusion in plantain fields would potentially retain 240,464 Mg more ecosystem carbon, comprising topsoil carbon plus tree biomass carbon, than standard farmer practice. Results demonstrate a potential “win–win scenario” where yield benefits, albeit modest, and conservation benefits can be obtained simultaneously. This could be considered as a transitional phase towards higher input use and thus higher yielding systems.

## Introduction

It is becoming more difficult and expensive to protect pristine tropical forest, so human-modified landscapes are increasingly important as a means of providing ecosystem services and conserving tropical biodiversity (Melo et al. 2013). The Congo Basin contains the second largest area of contiguous humid forest in the world and comprises the Democratic Republic of Congo (DRC), Republic of the Congo, Gabon, Equatorial Guinea, Central African Republic, and Cameroon. Smallholder farmers in this region use traditional slash-and-burn cultivation. Typically, an area of forest or fallow is manually cleared and the cut vegetation is left to dry. Farmers burn because they consider it the most labor-efficient method of clearing debris and they believe that it increases crop yields (Büttner and Hauser 2003). After a short cropping phase, the land is abandoned to fallow, then the cycle is repeated. According to the agronomic theory of Sébillotte (1985) the fallow has two distinct impacts: “l’effet précédent” (the preceding impact), what impact the fallow itself has on the plot conditions; and, “l’effet suivant” (the ‘following impact’), to what extent the following crop benefits from the changes made by the fallow.

Van der Werf et al. (2010) reported that Africa was the largest source of fire carbon dioxide over the period 1997–2009, contributing 52 % of global emissions. However, only approximately 15 % of African emissions were attributable to forest and deforestation fires; most emissions occurred in grassland, savanna, and woodland (i.e. woody savanna) (Van der Werf et al. 2010). For the forested Congo Basin, where individual land use patches are small and in a fine-scale mosaic, fire is difficult to detect and the biomass burned is hard to estimate (Eva and Lambin 2000) so this may lead to large errors in regional estimates. Indeed, Lauk and Erb (2009) reported that there are no data on the amounts of biomass burned in small vegetation fires. There are few prescribed burning or fuel reduction programs (Adams 2013), and fire use by smallholder farmers remains poorly understood (Carmenta et al. 2013). The few practicable alternatives to burning, such as improved fallows, tend to have low adoption rates, probably because of both their increased labor requirements (Snapp et al. 2002) and inconsistent effects on crop yield (Hauser et al. 2006). Labor-efficient alternatives demonstrating productivity increases need to be found.

The simplest alternative is not to burn yet to leave material to decompose in slash-and-mulch systems. Such systems are common in tropical America (Rosemeyer et al. 2000), and the Pacific Islands (Mertz et al. 2012), in areas where annual rainfall exceeds 2,000 mm per year and a lack of dry season precludes fire use (Vosti and Witcover 1996). Crops are planted through the mulch layer. This can be done without difficulty by forming a planting hole using a machete and so is particularly appropriate for crops propagated by corms or stem cuttings or those where multiple seeds are placed in the same pocket and inter-pocket spacing is relatively large, such as for maize. Tolstoy and DeBoer (1989) reported that farmers in NW Ecuador practice fire exclusion by choice because they believe that burning damages the soil. In the forest zone of Ghana, Quansah et al. (2001) observed that 11 % of farmers used slash-and-mulch, referred to in the Akan language as “proka”; to rot and add to soil. Recent surveys in the same region of Ghana revealed that 80 % of farmers report using “proka” as a soil fertility maintenance practice (Dawoe et al. 2012). However, there are few references to slash-and-mulch systems from the rest of Africa (Thurston 1997).

Fire exclusion may modify both the extent to which the following crop benefits from the fallow, and the succession of the future fallow and thus its impact on plot conditions. Combustion of biomass releases carbon (C) and converts part of the nutrient stock to ash, the remainder being lost by volatilization or fly ash (Giardina et al. 2000). Hölscher et al. (1997) estimated volatilization losses of nitrogen (N), phosphorus (P), and potassium (K) due to burning to be 96, 47, and 48 %, respectively, compared to amounts present in the previous aboveground biomass of a secondary forest in the Brazilian Amazon. Topsoil is modified first through the heat pulse and then through the effects of ash. Experimental heating of Nigerian soils demonstrated N and C losses at 200 °C and higher. Yet, availabilities of P, magnesium (Mg), iron (Fe), manganese (Mn), and zinc (Zn) were increased, if soil was heated to 100 °C, relative to a 25 °C control (Kang and Sajjapongse 1980). Ash generally increases soil pH through base addition. Ash adds basic cations to the soil and thus provides albeit temporary increases in topsoil pH (Giardina et al. 2000) compared to the previous forest. After field abandonment, soil pH tends to decline progressively as fallow ages given that the basic cations are being accumulated in the plant biomass. Aweto (1981) for Nigeria and McGrath et al. (2001) for Amazonia have demonstrated that older fallows have a lower soil pH than younger fallows.

Summarizing work in the Eastern Amazon, Denich et al. (2005) concluded that fire exclusion would address “the negative nutrient balances in slash-and-burn and the unburned slash would be a source of soil organic matter which would improve soil quality”. Comte et al. (2012), working in the Brazilian Amazon, showed that mulching rather than burning resulted in higher topsoil water holding capacity and total N. Hypothesized additional benefits of fire exclusion include the avoidance of negative impacts of burning on soil biota, a faster fallow and ecosystem carbon stock recovery, and a reduced wildfire risk. Yet, field operations such as planting and weeding might be more cumbersome due to debris and thus will increase labor requirements. Fire increases soil P availability in the short term but increases P sorption and thus reduces availability in the medium-term (Ketterings et al. 2002). Weed pressure might increase under fire exclusion due to higher seedbank viability (Szott et al. 1999). However, any impact of fire exclusion is likely to depend on both fallow age and field type so it needs to be tested across a range of systems.

Here we describe the common cropping systems and fallow dynamics in southern Cameroon. We assess, based on published work, which field types have a high potential propensity for fire exclusion. Synthesizing published and unpublished data from a series of experiments, we estimate what impact fire exclusion would have on crop yield, soil fertility, ecosystem carbon stocks, and fallow recovery under current fallow and reduced fallow scenarios. We also compare labor demands of the burned versus fire exclusion scenarios. By combining data on field type frequency and the average surface area of fields within the FMBA, we estimate the potential aggregate effects at the landscape level on production, landscape carbon, and labor requirements.

## Methods

### Experimental Area

The experimental sites were located within the humid Forest Margins Benchmark Area (FMBA) in southern Cameroon, an area of 1.54 million ha, selected over a south-to-north gradient of increasing human population and decreasing forest cover and thus divided into three equally sized blocks of low, medium, and high forest cover (Douthwaite et al. 2005). Rainfall varies from 1,350 to 1,900 mm p.a. in a bimodal distribution with much of the FMBA reported to experience 1,500 mm p.a. (Gockowski et al. 2004). Rainy seasons are from March to July and September to November. Soils are predominantly Ultisols (USDA classification).

**Fig. 1:**
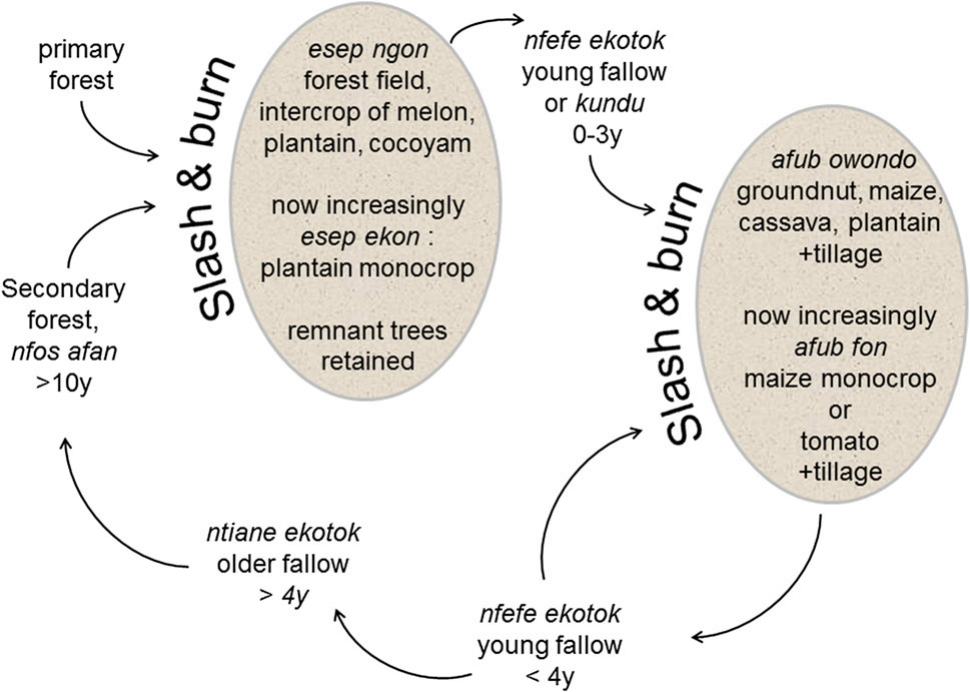
Food crop fields and fallows within the forest margin benchmark of central/southern Cameroon. Adapted after Guyer (1980), de Wachter (1997), Diaw (1997), Carrière et al. (2002), Russell and Tchamou (2001), Gockowski et al. (2004), Brown (2006)

Peoples within the FMBA belong to the Ewondo, Eton, Bulu, Bene, and Mvele sub-groups of the Beti, with the Ewondo being the most numerous. Figure 1 illustrates a generalized shifting cultivation system plus its evolution with increasing market orientation (adapted after Guyer 1980; de Wachter 1997; Diaw 1997; Carrière et al. 2002; Russell and Tchamou 2001; Gockowski et al. 2004; Brown 2006), illustrated using Ewondo terminology for fields after Guyer (1980) and for fallows after Diaw (1997). Forest fallow clearance is conducted during the long dry season (December–February), termed “esep”. However, traditionally, clearance is only partial as useful trees are retained (de Wachter 1997; Carrière et al. 2002). Carrière (1999), surveying approximately 10 ha of “esep” fields west of the FMBA, reported that, on average, 15 trees per ha greater than 40 cm diameter were retained. She counted 43 tree species, the most commonly encountered being *Terminalia superba*. In southern Cameroon, outside of the FMBA, Kanmegne (2004) reported that farmers retained 24 trees ha^−1^, on average. After partial clearance, the dried vegetation is flash-burned and shade-tolerant crops such as cucurbits (“ngon”, *Cucumeropsis mannii* or *Citrullus* spp.), plantain (*Musa* spp. AAB), and tannia (*Xanthosoma sagittifolium*) are planted in this “esep ngon” field, often by men, who can also conduct the weeding using a machete. After plantain harvest, the field is abandoned to a short fallow. This is later cleared, burned then hand-tilled and planted to an “afub owondo” field of groundnut (*Arachis hypogaea*), intercropped with cassava (*Manihot esculenta*), plantain, maize (*Zea mays*), and leafy vegetables as minor components. “Afub owondo” is usually under the operational control of women (Diaw 1997). In contrast to the “esep” fields, where any unburned biomass is left to rot, “afub owondo” fields are cleaned if the first burn is incomplete: women remove all partially burned material and weed debris, pile it around trunks of undesired large trees and conduct a second burn (Carrière 2003). After harvest, either the field is briefly fallowed (2–4 years) and the “afub owondo” is repeated, or it is fallowed for a long period, reaching young secondary forest growth and re-enters later the “esep” cycle. For both field types, the length of the cultivation period depends on the crop mixture employed and coincides with the harvest of the last crop: for example, this is usually plantain, which under traditional management can take from 1 to 3 years from planting to harvest of the mother plant (Norgrove and Hauser 2002b). However, it is generally difficult to specify when the cropping phase ends and the fallow begins as harvesting of plantains and tubers might continue after fallow vegetation has become established (Mutsaers et al. 1981). The right to clear a forest fallow for cultivation i.e. the “axe right”, is a male right (Veuthey and Gerber 2010) and clearance of the short fallow is usually conducted by men. Women are generally responsible for weeding although in certain field types, such as monocropped plantain and maize, from which produce is often sold, most agricultural tasks including weeding by machete, are done by men: women’s participation is limited (Gockowski and Ndoumbé 2004). Overall, the proportion of fields subject to burning is reportedly 94 % (Büttner and Hauser 2003), with little variation across the FMBA (Gockowski et al. 2004). Other important cropping systems within the FMBA include cacao (*Theobroma cacao*), estimated by Nolte et al. (2001) to occupy 100,000 ha, and home gardens (1,500 ha) (estimated from Nolte et al. 2001; Gockowski and Ndoumbé 2004). Cacao systems are usually established by farmers following an “esep” field. Both aforementioned systems are excluded here as they are long-term systems and therefore not subject to frequent fallowing and burning.

Fallows are generally assumed to be reducing in length, however, there are few data available on average fallow length (Ickowitz 2006). Russell and Tchamou (2001) reported that fallow lengths have halved in the middle FMBA block. In a recent global review (Van Vliet et al. 2012), including the assessment of changes in fallow length, two studies were cited from central Africa (Brown 2006; Bogaert et al. 2008), both of which showed a reduction in fallow length, and Brown (2006) worked specifically in the FMBA. As Fig. 1 demonstrates, there are two distinct fallow cycles and thus the overall average fallow length is also dependent on the proportion of fields entering each of the fallow cycles rather than just the duration of the fallow per se and so is difficult to calculate. Intensification and greater market orientation have modified this system and new, simplified field types are appearing. The traditional intercropped “esep ngon” field is in decline and is being replaced by the “esep ekon”, a simplified plantain monocrop field (Russell and Tchamou 2001). Other monocrop systems are also becoming more prevalent within the short fallow cycle, with maize, for fresh consumption, being the most common throughout the FMBA (Binam et al. 2004), although tomato monocrop is now the most important horticultural system in the northern FMBA block (Gockowski and Ndoumbé 2004). Furthermore, reduced fallow scenarios would involve a return to cultivation before four years for the “afub” cycle and a return to cultivation before the vegetation becomes secondary forest for the “esep” cycle (Fig. 1).

### Selection of Fields

**Fig. 2:**
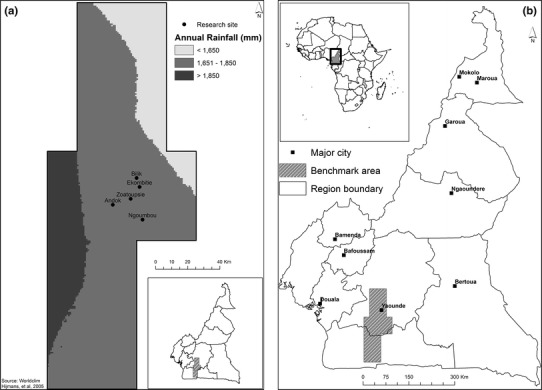
**a** Map of the forest margins benchmark area (FMBA) in Cameroon showing the distribution of research sites and rainfall. Annual rainfall averages (mm p.a.) for the sites during the experiments were 1634 (Bilik), 1533 (Zoatoupsie), 1533 (Ekombitié), 1786 (Andok), and 1836 (Ngoumbou). *Grayscale* represents the rainfall distribution according to data from the WorldClim database (Hijmans et al. 2005). **b** Situation of the FMBA in Cameroon and Africa

Of the field types described, some show a low propensity to fire exclusion. The major crop of the “afub owondo” is groundnut, which is traditionally tilled (Wendt 2002) for acceptable yields (Jordan et al. 2001), and there is incompatibility between the use of tillage and mulching (Erenstein 2003). Tomatoes are also grown after manual tillage, yields are much lower when not tilled (Tueche et al. 2013) and tillage would be difficult without prior burning of biomass. According to Brown (2004), maize is most commonly cultivated after 3–4 years of fallow and “esep” fields after 16–20 years of fallow. Selection of plots was according to the following criteria: being located within the FMBA in the central block, which has medium population density, medium forest cover and in areas with the most prevalent soils (Ultisols), and average rainfall in order to reduce confounding factors; no use of external inputs; access to original data; a randomized comparison of burning versus fire exclusion with at least four replications, with yield data. Thus, we selected the following experimental plots as representative of these field types: maize grown after 4 years of fallow (Hauser et al. 2008); plantain grown after 17 years of fallow (Norgrove and Hauser 2002a). Due to theft and a requirement to abandon the latter field, a field of plantain after 20 years of fallow was additionally included (Hauser and Mekoa 2009; Hauser et al. 2012a). Additionally, we selected plots with reduced fallow scenarios whereby maize was cropped after 2 years of fallow (Norgrove et al. 2003a, 2003b); and plantain after 6 years of fallow (Norgrove et al. 2003a, 2003b). Site locations were Andok, Ekombitié, Ngoumbou, Bilik, and Zoatoupsie, respectively (Fig. 2a, b). Rainfall was measured using rain gages read daily throughout the year either at the site itself or within 10 km of that site. Annual rainfall is given as the average over the cropping years (2 years for maize and 3 years for plantain) (Fig. 2a).

We used the plantain fields also to represent the traditional “esep ngon” (Fig. 1). Yields of plantain are unlikely to differ between the monocrop and intercrop system as ngon is low yielding; Kanmegne et al. (2007) reported ngon yields of 163 kg ha^−1^ and Phillip et al. (2009) showed that intercropping cucurbits with plantain had no negative yield effects.

### Experimental Design

All experiments selected had a randomized complete block design, including a burning versus fire exclusion factor, in 4 or 5 replications. Fallow vegetation was manually slashed with machetes and, for the longer fallows, some large trees were cut with a chainsaw. Cleared biomass was either burned (burned treatments) or was left in situ (fire exclusion treatments). Measures, such as complete removal of biomass and sweeping clean the soil surface, were used to avoid fire spreading between plots.

Short fallow sites were dominated by *Chromolaena* *odorata*, a pan-tropical invasive shrub that is now a dominant fallow species in much of West and Central Africa (Ngobo et al. 2004). This was cleared with machetes, left to dry then either burned or left in situ. The maize (*Zea mays* (L.) sub-species mays) used was a locally developed cultivar, CMS 8704, an open pollinated yellow-grained with a medium length to maturity. It was planted at 66,667 ha^−1^ without tillage or agrochemical inputs. Plots were manually weeded once during each growing period of the maize, as is local farmer practice and the plots were cultivated for two consecutive seasons. Maize stover was retained in situ after the first season and vegetation regrowth was slashed prior to replanting as before (Norgrove et al. 2003; Hauser et al. 2008).

Long fallow trials used plantain (*Musa* spp AAB) at 1,600 ha^−1^, a density comparable to that in “esep” fields estimated as 1,665 ha^−1^ by de Wachter (1997), and yield data were collected for 3 years. The 20-year-old system was cleared from secondary forest (Hauser et al. 2012a). The 17- and 6-year-old systems were abandoned *Terminalia ivorensis* plantations which had been established without any agrochemical use and had not received any management in the interim (Norgrove et al. 2003a, 2003b). At clearance, trees were retained, simulating traditional retention of trees by farmers: *Terminalia superba,* which has a similar growth form, is a commonly retained species in farmers’ esep fields in the region (Carrière 1999). Plot sizes were 15 × 15 m for the 20-year-old fallow site and 25 × 25 m for the 17- and 6-year-old fallow sites. The 17- and 6-year old plots were larger than normally required for agronomic trials as the growth of retained trees was monitored, necessitating sufficient number of trees per plot. Observations were made for 3 years except for the 17-year fallow site in which yields could not be measured due to theft. Trials were weeded manually with machetes approximately three times a year following farmer practice.

### Data Inputs for Yield, Soil Fertility, and Fallow Parameters

Data used for yield, soil fertility, and fallow parameters for maize and plantain systems under current and reduced fallow scenarios are summarized in Table 1. For maize after a 2-year fallow, leaf chlorophyll was measured at 28 and 56 DAP during the first cropping season using a Minolta SPAD hand-held meter. Readings were made on four plants per plot. One measurement was made in the center of the leaf on all leaves per plant and the mean per plant was recorded (Norgrove et al. 2003). The mean of the two dates is presented. This method was chosen as it is a reliable, fast, and non-destructive way to measure directly leaf chlorophyll and thus estimate relative plant nitrogen (N) status (Costa et al. 2001).

**Table 1:** Summary of datasets used

C/R	Y/S/F		Burned	Fire exclusion	*P*	Ref^c^	Use of 1/*x*
		Short fallow cycle maize					
C4	Y	Dry grain yield (Mg ha^−1^)	1.12	1.18	ns	Hauser et al. (2008)	
C4	S	pH (0–5 cm depth)	4.13	4.41	ns	Hauser et al. (2008)	
C4	S	Total N (0–5 cm depth) (mg g^−1^)	1.78	1.98	ns	Hauser et al. (2008)	
C4	S	Avail P (0–5 cm depth) (mg Kg^−1^)	10.1	13.2	ns	Hauser et al. (2008)	
C4	S	Organic C (0–5 cm depth) (mg g^−1^)	24.7	28.5	ns	Hauser et al. (2008)	
R2	Y	Dry grain yield (Mg ha^−1^)	1.70	2.90	*	Norgrove et al. (2003a)	
R2	Y	Leaf chlorophyll (SPAD)	30.5	34.8	*	Norgrove et al. (2003a)	
R2	S	pH (0–5 cm depth)	6.73	6.69	ns	Norgrove et al. (2003a)	
R2	S	Maximal soil temperature at 20 mm depth (°C)	29.8	27.3	*	Norgrove et al. (2003b)	*x*
R2	S	Earthworm casts prior to fallowing (Mg ha^−1^ year^−1^)	1.59	1.71	ns	Norgrove et al. (2003b)	
R2	S	Decomposition of mulch residues (half-life) days	81.7	44.1	*	Norgrove et al. (2003b)	*x*
R2	S	Bulk density (Mg m^−3^) (0–5 cm depth)	0.94	0.85	*	Norgrove et al. (2003b)	*x*
		Long fallow cycle plantain					
C20	Y	Plantain fresh bunch yield (Mg ha^−1^)	6.26	6.16	ns	Hauser et al. (2012)	
C20	Y	Planting to harvest time	921	923	ns	Hauser et al. (2012)	*x*
C20	S	pH (0–10 cm depth)	5.09	4.73	ns	Hauser et al. (2012)	
C20	S	Total N (0–10 cm depth) (mg g^−1^)	2.59	2.43	ns	Hauser et al. (2012)	
C20	S	Avail. P (0–10 cm depth) (mg kg^−1^)	10.48	6.90	ns	Hauser et al. (2012)	
C20	S	Organic carbon (0–10 cm depth) (mg g^−1^)	27.3	26.4	ns	Hauser et al. (2012)	
C20	F	Tree seedlings in understory, end of crop phase^a^ (kg ha^−1^)	7.08	26.67	*	Hauser and Mekoa (2009)	
C17	S	Maximal soil temperature at 60 mm depth (°C)	26.9	26.5	ns	Norgrove and Hauser (2000)	x
C17	S	Earthworm cast production (Mg ha^−1^ year^−1^)	27.6	25.9	ns	Norgrove and Hauser (2000)	
C17	S	Organic C (0–10 cm depth) (mg g^−1^)	18.0	17.2	ns	Norgrove and Hauser (unpubl.)	
C17	S	pH (0–10 cm depth)	5.11	4.78	ns	Norgrove and Hauser (unpubl.)	
C17	S	Total N (mg g^−1^) (0–10 cm depth)	1.62	1.55	ns	Norgrove and Hauser (unpubl.)	
C17	F	% Remnant trees remaining after burn	88	100	*	Norgrove and Hauser (unpubl.)	
C17	F	Remnant tree growth (kg tree^−1^ year^−1^)	158	100	*	Norgrove and Hauser (unpubl.); Norgrove and Hauser (2002a)	
R6	Y	Plantain fresh bunch yield (Mg ha^−1^)	8.4	9.7	*	Norgrove and Hauser (2002b)	
R6	Y	Planting to harvest time (days)	671	560	*	Norgrove and Hauser (2002b)	*x*
R6	S	Maximal soil temperature at 60 mm depth	30.2	29.1	*	Norgrove et al. (2000)	*x*
R6	S	Earthworm cast production (Mg ha^−1^ year^−1^)	51.4	39.6	ns	Norgrove and Hauser (unpubl.)	
R6	S	Organic C (0–10 cm depth) (mg g^−1^)	20.4	23.2	*	Norgrove and Hauser (unpubl.)	
R6	S	pH (0–10 cm depth)	5.15	4.94	ns	Norgrove et al. (unpubl.)	
R6	S	Total N (0–10 cm depth) (mg g^−1^)	1.87	1.98	ns	Norgrove et al. (unpubl.)	
R6	S	Decomposition of weed residues (^1^/_2_ life) (days)	46.9	41.1	*	Norgrove et al. (2000)	*x*
R6	F	% Remnant trees remaining after burn	63	100	*	Norgrove and Hauser (unpubl.)	
R6	F	Remnant tree growth (kg tree^−1^ year^−1^)	128	154	*	Norgrove and Hauser (unpubl.); Norgrove and Hauser (2002a)	
R6	F	Tree seedlings in understory, end of crop phase^b^ (kg ha^−1^)	20	86	*	Norgrove et al. (unpubl.)	

*P* refers to the significance of a difference between burned and fire exclusion treatments and is annotated as * for *P* < 0.05 and ns for *P* > 0.05

The use of the reciprocal, 1/*x*, was for parameters where a lower value denoted an improvement

*C* Current fallow length, *R* reduced fallow length: 2, 4, 6, 17, 20 are respective fallow lengths (years), *Y* yield parameters, *S* soil fertility parameters, *F* fallow regeneration parameters.

^a^ At 942 days after planting (DAP);

^b^ at 966 DAP

^c^References for further details, however, actual data values have not been presented in these papers as they incorporated other treatments

In the 2-year fallow site, plots were harvested at 105 DAP in the first season and 98 DAP in the second season and in the 4-year fallow site, harvests were between 95 and 105 DAP. Maize dry grain yields were estimated through plot harvests, grain separation, and dry matter determination (Norgrove et al. 2003a; Hauser et al. 2008). Plants were counted, cobs were removed, counted, husked, and weighed fresh. A subsample of cobs was taken and fresh weight was recorded. They were then dried at 65 °C to constant mass and the grain dry matter yield was determined by weighing the seeds after shelling the cobs (Hauser et al. 2008).

In the long fallows, plantain yield refers to whole bunch mass on a per hectare basis, harvested when ripe. The time from planting to harvest was recorded for each plant (Norgrove and Hauser 2002; Hauser et al. 2012). Decomposition rates of plant residues can be used as an indicator of ecosystem functioning (Hauser et al. 2005). In the maize after a 2-year fallow, decomposition of slashed fallow vegetation during the cropping season was estimated in plots that were mulched and in additional plots subjected to burning that were not used for the yield assessment reported here (Norgrove et al. 2003). In the plantain after a 6-year fallow, decomposition rates of leaf material of *C. odorata,* the dominant weed residue, were measured after two weedings (Norgrove et al. 2000). Less fluctuating soil temperatures promote soil biota activity and nutrient cycling and can reduce the rate of fallow seedbank decline (Gallagher et al. 1999). In the maize after a 2-year fallow, and the plantain after 6- and 17-year fallows, soil temperature readings were made twice per week at 20 or 60 mm depth between 14.00 and 14.30 h when temperature is assumed maximal (Norgrove et al. 2000, Norgrove et al. 2003b; Norgrove and Hauser 2000). Earthworm surface cast production can be used as a rough indicator of soil quality albeit with limitations (Hauser et al. 2012b). Earthworm casts were collected throughout the maize seasons and in the final year of plantain cultivation they were collected twice per week from four 0.5 m × 0.5 m frames per plot in the 6- and 17-year fallow sites (Norgrove et al. 2003b; Norgrove and Hauser unpublished; Norgrove and Hauser 2000). Casts were oven-dried to constant mass, dry masses recorded and expressed on a per hectare basis.

In all experiments, soil sampling was conducted post-burning. In the 2-year fallow, six undisturbed topsoil (0–5 cm depth) samples per plot were taken at the end of the first cropping season using 100 cm^3^ cores of 5.0 cm diameter and 5.0 cm length. In the 4-year fallow, samples were taken with a standard 2 cm diameter auger. For the 6- and 17-year fallows, soil was sampled 4 weeks after burning. Nine samples were taken per plot comprising each of five auger insertions at 0–10 cm depth and three core samples at 0–5 and 5–10 cm for bulk density determination (Norgrove and Hauser unpublished). For the 20-year fallow, 9 samples per plot were taken (Hauser et al. 2012a, b). All samples were dried to constant mass and were weighed. Auger samples were passed through a 2 mm sieve and analyzed for organic C, determined by chromic acid digestion and spectrophotometric procedure (Heanes 1984). Soil pH was determined by water suspension at a 1:5 ratio. Total N was determined using the Kjeldahl method for digestion and ammonium sensitive electrode determination (Bremner 1982; Bremner and Tabatabai 1972). Available *P* was determined by the Mehlich-3 procedure (Mehlich 1984). Bulk density, where applicable, was calculated as the dry mass of the soil core divided by the volume. To estimate soil carbon amounts, soil organic carbon was multiplied by bulk density of the corresponding sample.

For the 20-year fallow, understory vegetation biomass was sampled at the end of the cropping phase and the % biomass contribution of tree seedlings was calculated (Hauser and Mekoa 2009). For the 6- and 17-year fallows, remnant tree growth was recorded by measuring breast height girth (bhg) with a tape measure (Norgrove and Hauser 2002a). As *Terminalia ivorensis* has a circular cylindrical, non-tapered bole, bhg was converted to surface area (*A*_s_) using the following equation: *A*_s_ = (gbh)^2^/4π. Total dry mass per tree was estimated according to a regression equation specifically developed at this site by :Deans et al. 1996 Tree dry mass (Mg) = 0.763*A*_s_ (m^2^), *r* ^2^ = 0.985, *n* = 6. Any tree that died from the burn was also recorded. In the 6-year fallow, 12 understory vegetation samples, 0.5 m × 0.5 m, were taken per plot at the end of the 3-year cropping phase, divided by species. Samples were dried, dry masses recorded (Norgrove et al. unpublished) and tree seedling mass (kg ha^−1^) calculated.

### Statistical Analysis and Data Conversion

Data were analyzed in SPSS v22 using a blocked, one factorial generalized linear model at two levels, burning versus fire exclusion, and using a significance level of *P* < 0.05. We conducted exploratory analysis on all data to assess if sample variances were correlated with the means and we transformed data to stabilize the variance, if necessary. Chlorophyll data and tree growth data were square-root transformed prior to analysis as is appropriate when the mean is proportional to the standard error (Sokal and Rohlf 1995). Data expressed as percentages (remnant trees burned, mulch mass remaining) were transformed such that *Y* = arcsine (sqrt *y*) where 0 < *y* < 1 as is appropriate for proportions (Sokal and Rohlf 1995). Other data analyses were performed on untransformed data.

To allow us to compare the magnitude of change of different parameters, we expressed our results as the percentage change with fire exclusion relative to the burned “farmer practice” case such that % change = (fire exclusion − burned)/burned*100 %. For parameters for which a lower numerical value signifies an improvement in soil conditions, we expressed the parameter in the reciprocal, 1/*x*: for decomposition rates; 1/half-life; 1/maximal soil temperature; 1/bulk density; 1/time from planting to harvest.

We estimated tree growth and carbon accumulation (assuming 45 % C in biomass) in remnant trees in a typical “esep” by multiplying our per-tree estimates by the average density of 15 trees per ha found in “esep” fields in the region (after Carrière 1999). We used a 3 year crop phase and assumed either a 6-year fallow in the case of the field continuing to a reduced fallow “esep” cycle or a 2-year fallow if continuing to a reduced fallow “afub owondo” cycle.

### Labor and Profitability Estimates

For the reduced fallow scenarios (2-year maize and 6-year plantain), we estimated relative labor inputs. We defined the components of labor input as the sum of clearance, burning and other land preparation, paring (plantain only), planting, weeding, and harvesting. For the 2-year maize, we based our calculations on published estimates from Tonye et al. (1997), who conducted a similar assessment within the FMBA, supplemented with data on clearance labor from Gockowski et al. (2004). For the 6-year fallow, we used data from Hauser (2007) for labor requirements of paring the plantain suckers. We directly measured the labor requirement of manual weeding by allocating teams of two individuals to each replicate and timing. The labor requirements of other tasks were estimated after Gockowski et al. (2004). All labor estimates were based on a 6-h day. To estimate partially the profitability, we used a value of the opportunity cost of labor of US$ 2 day^−1^ (after Bellassen and Gitz 2008) and identical to that used by Tonye et al. (1997). This is higher than US$ 1.21 used by Gockowski et al. (2001). For farm-gate prices, we used data from FAO (2011) of US$ 407.6 Mg^−1^ for maize grain and US$ 363.4 Mg^−1^ for plantain bunches. We modified data on average field size and prevalence from farmer surveys of Gockowski et al. (2004) by separating their “horticulture” class into tomato, maize, and plantain systems, following Gockowski and Ndoumbé (2004). We combined these data with remote sensing results of Nolte et al. (2001) to estimate the areas occupied by different field types within the FMBA. To obtain estimates of production differences, we multiplied differences in yield, soil organic carbon amounts, and carbon stocks in remnant trees by these estimated field areas.

## Results

**Fig. 3:**
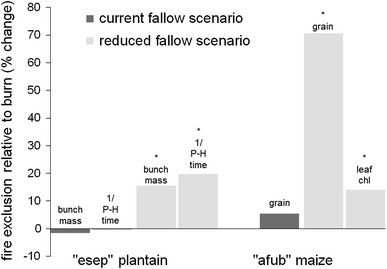
Relative (%) changes in yield and yield components of plantain and maize with fire exclusion under current (20-year plantain, 4-year maize) and reduced fallow scenarios (6-year plantain, 2-year maize). *Denotes significankt difference (*P* < 0.05) between fire exclusion and burning treatments

**Fig. 4:**
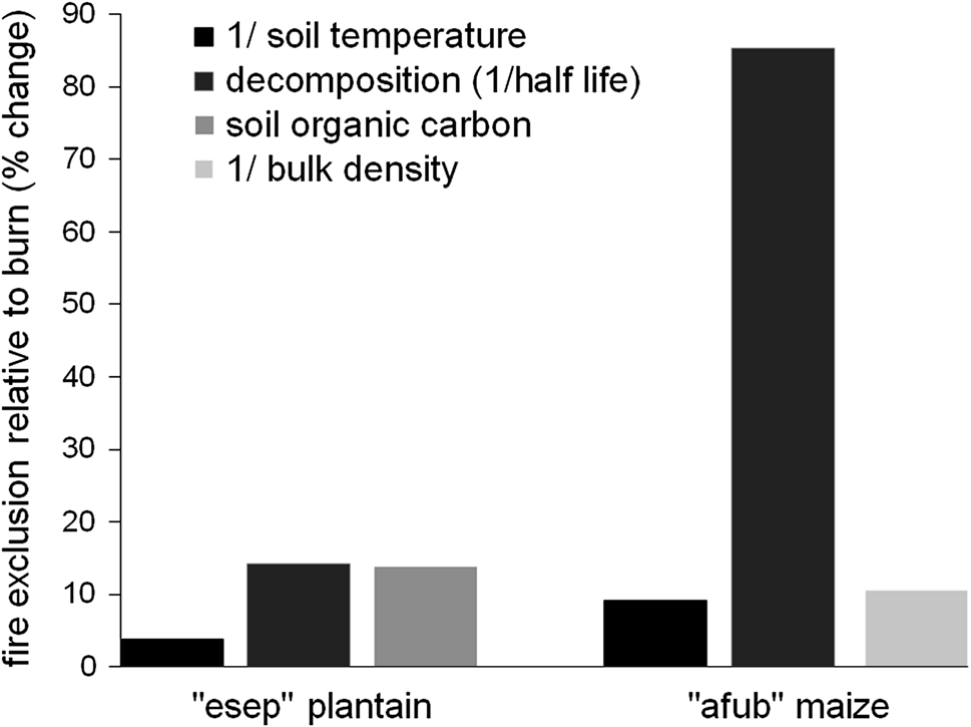
Relative (%) changes in those soil fertility parameters where significant (*P* < 0.05) differences were detected between fire exclusion and burning treatments. All were from reduced fallow scenarios (6-year plantain, 2-year maize). Soil temperature at 60 and 20 mm depth for plantain and maize, respectively. Decomposition (1/half-life) for weed residues in plantain plots and incubated mulch in maize plots

**Fig. 5:**
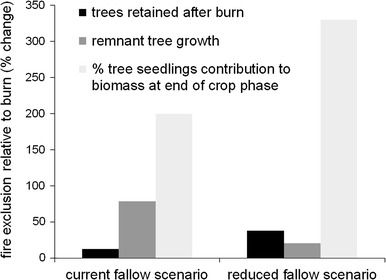
Relative (%) changes in fallow recovery parameters in “esep” fields under current (20-year, 17-year fallows) and reduced (6-year) fallow scenarios. *Denotes that parameter is significantly different (*P* < 0.05) between fire exclusion and burning treatments

Under current fallow scenarios, we found no significant differences in yield (Fig. 3) or in soil fertility parameters between burned and fire exclusion treatments. For the reduced fallow scenarios, we found that fire exclusion resulted in significantly higher yields of maize (71 % more grain and 14 % higher chlorophyll levels in leaves) and plantain (115 % higher bunch yield and 20 % shorter time from planting to harvest) (Fig. 3). Under the reduced fallow scenarios, fire exclusion and retention of surface mulch maintained lower topsoil temperatures, promoted faster decomposition, lowered soil bulk density, and increased soil organic carbon concentrations, relative to initial burning (Fig. 4). However, differences in soil pH and total N were not detected. The response of earthworm cast production was generally insignificantly different between fire exclusion and burned treatments. For all plantain fields with fire exclusion, we found positive effects on the growth of remnant trees, and the proportion of tree seedlings remaining in fallow, suggesting that fallow recovery rates would be enhanced (Fig. 5).

For the reduced fallow scenario, we found that fire exclusion led to an approximately 50 % increase in labor requirements for planting, weeding, and harvesting both in the maize and plantain systems, compared with the farmer practice of burning. This estimated labor demand increase was 34 days ha^−1^ for the maize and 40 days ha^−1^ for the plantain. Ceteris paribus, applying farm-gate prices to the yield differences between the burned and fire exclusion treatments of 1.2 Mg ha^−1^ in the case of maize and 1.3 Mg ha^−1^ for the plantain, we estimated an increased revenue of 489 and 472 US$ ha^−1^, respectively, or 421 and 388 US$ ha^−1^ after the opportunity cost of extra labor had been deducted.

**Fig. 6:**
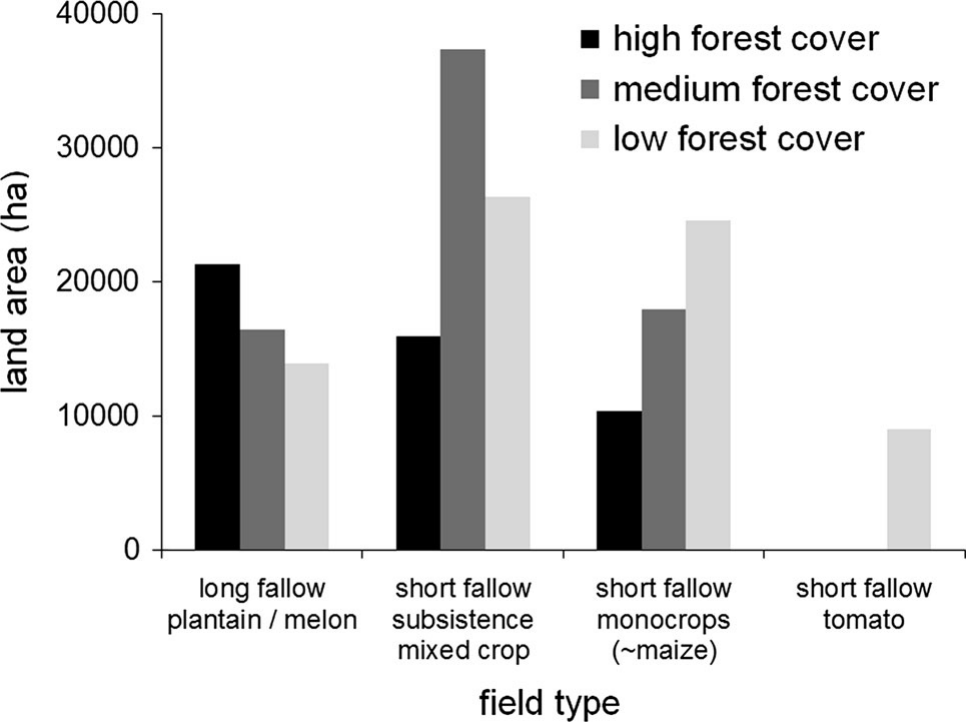
Estimated areas (ha) of pooled “esep” long-fallow fields and three types of short-fallow fields in the FMBA, separated by forest cover block. Calculated after Nolte et al. (2001), Gockowski and Ndoumbé (2004), Gockowski et al. (2004)

Figure 6 shows the extent of the grouped “esep” systems and the three groups of short fallow systems in the three FMBA blocks. Applying yield differences to the land areas under “esep” and maize monocrop under a reduced fallow scenario would result in potential increased production of 27662, 21384, and 18128 Mg of plantain and 12474, 21580, and 29474 Mg of maize in the high, medium, and low forest cover blocks, respectively. Potential carbon “savings” or higher retention across the entire FMBA calculated from the application of fire exclusion to the “esep” fields only, amounted to, potentially, 159,043 Mg of soil carbon at 0–10 cm depth. If the land were returned to the “esep” cycle after a 6-year fallow, 81,421 Mg of tree biomass carbon would be retained in the growth of remnant trees over the crop and fallow period. If the land were returned to the “afub” cycle after a 2-year fallow, then 45,234 Mg of carbon would be retained in the growth of remnant trees over the crop and fallow period, giving total (topsoil plus remnant trees) of 240,464 and 204,277 Mg carbon, respectively.

## Discussion

Under current fallow scenarios, we found no evidence for any yield advantage of fire exclusion over farmer practice, nor detectable effects on soil fertility parameters. While we did not estimate labor inputs for these scenarios, requirements are likely to be higher for the fire exclusion practice. However, there was evidence in the “esep” systems, even under the current fallow scenario, of an improvement in fallow recovery indicators implying that productivity could be increased with fire exclusion. In line with this finding, under the reduced fallow scenarios, improved soil fertility, and fallow recovery indicators were obtained as well as higher yields. Therefore, fire exclusion might compensate, to some extent, for reduced fallow lengths by allowing recovery within a shorter period and avoiding further degradation. This concurs with Styger et al. (2007), who found, based on farmer interviews and perception of the system, that agricultural fire was a main driver of degradation in Madagascar.

According to Kato et al. (1999), fire exclusion is likely to be more advantageous with longer cropping phases, however, we found a greater positive yield impact, given as the percentage yield change, on the 3-month maize than the 3-year plantain. Cereals such as maize have a high nitrogen demand, and, indicated by the higher chlorophyll levels found here, plant nitrogen status was improved, given expected reduced N loss (Hölscher et al. 1997) with fire exclusion. The non-significance of effects in older fallows may be partially because the higher spatial variability in older systems makes effects more difficult to detect. Other reasons for a lack of discernible effects could be that soil fertility levels are sufficiently high to mask any benefit from biomass retention. Both plantain and maize can show positive responses to mulch layers, whether applied or retained; Lal (1997) reported that the application of non-incorporated mulch consistently produced higher yields of maize in southern Nigeria. Norgrove and Hauser (2014), reviewing all research on plantain conducted in West and Central Africa, concluded that mulch application should be universally recommended. Thus, our results concur with those from a broader analysis and thus appear representative for the region. Furthermore, rainfall during the cultivation period at the shortened fallow sites was close to the long-term average for the region.

Here, the measured labor increase due to weeding in fire exclusion plots after a 6-year fallow was in spite of their lower weed biomass (Norgrove et al. 2000). We explain the increased labor requirement with fire exclusion by the difficulty of moving around the field and conducting operations when the land is covered with debris, supporting the farmers’ views on burning to reduce labor, regardless of fallow length. While farmers may initially be reluctant to adopt any system with increased labor demands, under the reduced fallow scenarios, the fire exclusion system provided higher returns than burning, for both crops, when labor requirements and farm-gate prices were accounted for. One potential drawback of an increased labor requirement in the maize system is that the FMBA has a bimodal rainfall distribution with rainy seasons of approximately 3 months each. Crops requiring 3 months to mature, such as maize, need to be planted immediately at the onset of the rains and farmers are aware of this requirement. Any increase in time required for planting maize may be critical given a peak in labor demand at the start of the season. In our analysis, we used a seasonally constant opportunity cost (after Tonye et al. 1997, Bellassen and Gitz 2008) and White et al. (2005) have discussed the limitations of this approach. Yet, based on our local knowledge, the value used is comparable to the wage offered within villages at peak labor periods. For plantain, given that it is perennial, planting can be more easily staggered to take into account labor availability with a lower risk of yield loss, so is therefore less sensitive to labor peaks. Southern Cameroon has also experienced net migration from urban to rural areas as a response to economic crisis and unemployment in cities (Sunderlin et al. 2000), and this increases rural labor availability.

A limitation of our study is that we are assuming ceteris paribus, that all other things remain equal and results are not affected by technology adoption. We have also assumed linearity in the variable response with scale, in line with other authors who have estimated carbon in tropical forests in Africa from plot data (for example, Lewis et al. 2009). The linearity assumption can lead to inaccuracies, for example, for labor inputs, economies of scale tend to arise when moving from plots to field sizes. However, in this region of Cameroon, existing fields of these types are small, between 0.28 and 0.35 ha (Gockowski et al. 2004). Thus scaling-up from plots is less problematic. Other possible sources of error include yield heterogeneity in plots not being captured by sampling methods. Here, however, we mainly relied on whole plot harvests thus this is taken into account.

There is indeed little information on African carbon stocks with few ground truthing data which could be used to calibrate those data obtained through remote sensing (Ciais et al. 2011). Cloud cover plus the fine mosaic nature of land use systems in the Congo Basin make data collection and interpretation difficult. Such shifting cultivation systems are not represented in the millennium village project (i.e. Deckelbaum et al. 2006) and were also missing from a recent paper on African farming systems (Vrieling et al. 2011). Thus, data on both ecosystem carbon changes and the dynamics of these farming systems are scant. This paper thus provides information on the potential biophysical possibilities. Adjusting farming systems to successfully incorporate any technology change and a desirable end result both in terms of farmers’ livelihoods and broader ecosystem carbon stock maintenance requires further considering and encompassing institutional, political, socio-economic factors.

In the Congo Basin, given endemic poverty, livelihood improvement should be the top policy priority. At the same time, at the global level, there is a debate about how agricultural intensification in the humid tropics will affect conservation. Gockowski and Sonwa (2011) argue that the main driver of deforestation in West and Central Africa has been the expansion of extensive smallholder agriculture and that, had intensification options, such as fertilizer application for cacao, been introduced in the 1960s then much deforestation would have been avoided. On the other hand, Phelps et al. (2013), referring to DR Congo, suggest the contrary, i.e. the occurrence of Jevons’ paradox, that farmers will clear more land when yields increase as it becomes more profitable to do so. The data synthesized in the current paper illustrate modest yield increases with fire exclusion and could be viewed as a transitional phase towards higher input use and thus higher yielding systems. Encouraging farmers to forgo burning their fields may be problematic yet the negative impacts of fire on livelihoods are already locally understood. Sassen and Jum (2007), working in a village in Central Cameroon to assess views of locals of threats to ecosystem services, reported that villagers ranked fire as the second greatest threat (mentioned by 60 % of households) after tree felling. Norgrove et al. (2003a), in southern Cameroon, reported that during a single dry season 15 % of young cacao agroforests in a participatory experiment were destroyed by fires that had “escaped” from neighbors’ plots. At that time, farmers admitted that they had neither the finance nor the recourse to take any legal action. If fire exclusion scenarios were followed for the two field types here, the number of fire events would be greatly reduced. The risk of wildfires is particularly high in the low forest cover block as fire-prone short fallows occupy 22 % of that land area, compared with only 8 and 4 % in the medium and high forest cover blocks (Nolte et al. 2001). Biomass of shorter fallows is more susceptible to fire as it usually has a high proportion of relatively flammable grasses and tends to dry out faster and more completely during the dry season (Sorrensen 2000). However, humid forests are also susceptible to fire risk in seasonal climates: Bucini and Lambin (2002) reported that fires affected 5.8 % of the humid forest in the south of neighboring Central African Republic. Our results could be applicable to much of the humid forest zone of West and Central Africa (sensu Jalloh et al. 2012), ranging from Guinea Bissau in the west to the Democratic Republic of Congo (DRC) in the south-east. Plantain is one of the most important crops in this region (summarized by Norgrove and Hauser 2014) and almost all is grown under “low resource” conditions by smallholder farmers (Altieri et al. 2012). In West and Central Africa, maize is grown both in moist savanna and humid forest zones. It occupies 21 % of the area devoted to cereals and mean annual consumption is 43 kg per capita (Pingali 2001). Fallows are reported to be shortening in this region and elsewhere in the humid tropics: Van Vliet et al. (2012) reported in a global review of shifting cultivation systems, 49 out of 59 studies reviewed reported declining fallow lengths.

An alternative strategy would be a switch to perennial tree and palm crops. This would also reduce fire hazard, as, although such systems are usually established through slash-and-burn, fire is not commonly used during the extended life cycle of the crop. Yet, farm households are unlikely to reduce the production of annual food crops for family consumption. According to FAO, there have indeed been increases in land area under cacao and oil palm, yet decreases in land area under coffee in Cameroon, as a whole. However, the increase in land area under cacao has been much less than the increase in land area under maize: in 2003, areas under maize and cacao were roughly similar. By 2012, area had increased by 65 % for cacao yet by 135 % for maize (FAOSTAT 2011, accessed September 2014).

Fire exclusion practices, retaining a mulch layer, could potentially pose a fire risk in itself. However, in the humid tropics, this is unlikely to be the case as mulch will be humid for much of the growing season and the layer will be greatly reduced during the following dry season. On the negative side, farmers often state that burning kills snakes and rodents (Quansah et al. 2001 for Ghana) and, on the contrary, mulch layers may harbor such species and pose a risk to humans. Yet, while the role of mulch as a hide-out for rodents and their predators, snakes, is often mentioned, no published controlled studies on its impact have been found. Schill et al. (2000) reported that farmers feared that mulching of plantain increased crop pest incidence. However, studies have shown that either this is not the case or that mulching increases yields to such an extent that the benefit outweighs the cost (Norgrove and Hauser 2014).

We did not assess fallow recovery indicators in the short fallow maize systems. However, the exclusion of fire at the “esep” stage may have residual benefits for the following short fallow fields (“afub owondo”). To align conservation and production goals, it is essential that sufficient fields are retained in the longer fallow “esep” cycle thus ensuring forest fallows in the landscape albeit with a reduced fallow length. This also prevents a potential shift in labor allocation from men to women; men are responsible for clearing (Veuthey and Gerber 2010) and women usually are responsible for weeding and harvesting (Guyer 1984). As weeding is generally easier in fields cleared after older fallows (Dvořák 1992), shifting more fields into the shorter cycle would also increase female workload.

## Conclusion

Our results demonstrate a potential “win–win scenario” where yield benefits, albeit modest, and conservation benefits, such as faster fallow recovery and increased ecosystem carbon stocks, can be obtained simultaneously. Fire exclusion practices applied in maize and plantain systems could be considered as a transitional phase towards higher input use and thus higher yielding systems.
